# Choline Kinetics in Neonatal Liver, Brain and Lung—Lessons from a Rodent Model for Neonatal Care

**DOI:** 10.3390/nu14030720

**Published:** 2022-02-08

**Authors:** Wolfgang Bernhard, Marco Raith, Anna Shunova, Stephan Lorenz, Katrin Böckmann, Michaela Minarski, Christian F. Poets, Axel R. Franz

**Affiliations:** 1 Department of Neonatology, University Children’s Hospital, Tübingen University Hospital, 72076 Tübingen, Baden-Wuerttemberg, Germany; anna.shunova@med.uni-tuebingen.de (A.S.); Stephan.lorenz@ofld.de (S.L.); katrin.boeckmann@med.uni-tuebingen.de (K.B.); Michaela.minarski@med.uni-tuebingen.de (M.M.); Christian-F.Poets@med.uni-tuebingen.de (C.F.P.); Axel.Franz@med.uni-tuebingen.de (A.R.F.); 2 Max-Planck-Institut für Psychiatrie, 80804 Munich, Bavaria, Germany; Marco_raith@psych.mpg.de; 3 Center for Pediatric Clinical Studies, University Children’s Hospital, Tübingen University Hospital, 72076 Tübingen, Baden-Wuerttemberg, Germany

**Keywords:** betaine, brain, choline, phospholipids, liver, lung, preterm infant, stable isotope labelling, tandem mass spectrometry

## Abstract

Choline requirements are high in the rapidly growing fetus and preterm infant, mainly serving phosphatidylcholine (PC) synthesis for parenchymal growth and one-carbon metabolism via betaine. However, choline metabolism in critical organs during rapid growth is poorly understood. Therefore, we investigated the kinetics of D9-choline and its metabolites in the liver, plasma, brain and lung in 14 d old rats. Animals were intraperitoneally injected with 50 mg/kg D9-choline chloride and sacrificed after 1.5 h, 6 h and 24 h. Liver, plasma, lungs, cerebrum and cerebellum were analyzed for D9-choline metabolites, using tandem mass spectrometry. In target organs, D9-PC and D9-betaine comprised 15.1 ± 1.3% and 9.9 ± 1.2% of applied D9-choline at 1.5 h. D9-PC peaked at 1.5 h in all organs, and decreased from 1.5–6 h in the liver and lung, but not in the brain. Whereas D9-labeled PC precursors were virtually absent beyond 6 h, D9-PC increased in the brain and lung from 6 h to 24 h (9- and 2.5-fold, respectively) at the expense of the liver, suggesting PC uptake from the liver via plasma rather than local synthesis. Kinetics of D9-PC sub-groups suggested preferential hepatic secretion of linoleoyl-PC and acyl remodeling in target organs. D9-betaine showed rapid turnover and served low-level endogenous (D3-)choline synthesis. In conclusion, in neonatal rats, exogenous choline is rapidly metabolized to PC by all organs. The liver supplies the brain and lung directly with PC, followed by organotypic acyl remodeling. A major fraction of choline is converted to betaine, feeding the one-carbon pool and this must be taken into account when calculating choline requirements.

## 1. Introduction

### 1.1. Impact of Choline Metabolism on Preterm Infant Development

Choline is an essential nutrient and constitutive tissue component, with high and tightly regulated concentrations, particularly in the parenchyma. Requirements are high in the fetus and preterm infant due to their 3–4-fold higher physiologic growth rate compared to the term born. Therefore, placental choline transfer is high, and plasma choline is 30–50 µM in the fetus vs. 10–17 µM in pregnant women [1]. In preterm infants’ plasma, however, choline untimely drops by ≥50% within 48 h after birth [2]. This potentially affects parenchymal growth because the synthesis of phosphatidylcholine (PC), the main and highly concentrated choline-containing phospholipid of all parenchyma and many secretions, depends on choline plasma concentration [3,4,5]. This is due to the high Michaelis constants (K_M_) of ubiquitous choline transporters (CTL2-4) [6,7].

In adult mice, choline deficiency causes its redistribution from the lungs to the liver [8], potentially affecting lung tissue integrity. The physiologic kinetics of choline and its metabolites in neonatal organisms is, however, largely unknown. All organs depend on choline supply and have tightly regulated concentrations of PC and sphingomyelin (SPH), the second choline-containing phospholipid. They comprise about 50% and 8% of tissue phospholipids and account for 85–90% of total choline pools [6,9]. However, their concentrations in immature relative to mature organs are not clear but influence the degree of the cumulative deficit during postnatal [1,4].

Understanding the physiologic kinetics of choline and its metabolites is of major clinical relevance to the development of the liver, lung and brain, the most critical organs in preterm infants. The liver is central to choline/PC metabolism and choline depletion leads to steatosis and liver failure in adults. Similarly, cholestasis, which is frequently encountered in ill preterm infants, may be related to choline deficiency as well [10,11]. At least 50% of the hepatic PC pool recycles daily via bile and the enterohepatic cycle [12,13]. Additionally, the liver releases free choline and betaine, and PC via very-low-density lipoproteins (VLDL, which comprise ~20% PC), into plasma, thereby supplying other organs. In plasma, PC of lipoproteins is the main transporter of arachidonic (ARA, C20:4-n6) and docosahexaenoic acid (DHA, C22:6-n3) [14]. In the adult lung, PC concentration is similarly high as in the liver. The majority of its synthesis by type-II-pneumocytes is for basolateral secretion, contributing to systemic choline homeostasis via PC transfer to apolipoprotein A1 and high-density lipoprotein (HDL, ~25% PC) formation [15,16]. There is little information on whether this applies to (preterm) infants. During choline deficiency, a net reversal choline/PC transport from the lung to the liver [8] may cause pulmonary choline exhaustion, potentially contributing to impaired lung development. Finally, the cerebrum and cerebellum are rich in PC and SPH as well, so cumulative choline deficiency during development may impair neurodevelopment, even in the absence of overt brain lesions [1,4,17,18,19,20].

### 1.2. Targeting Choline Metabolism

**Figure 1:**
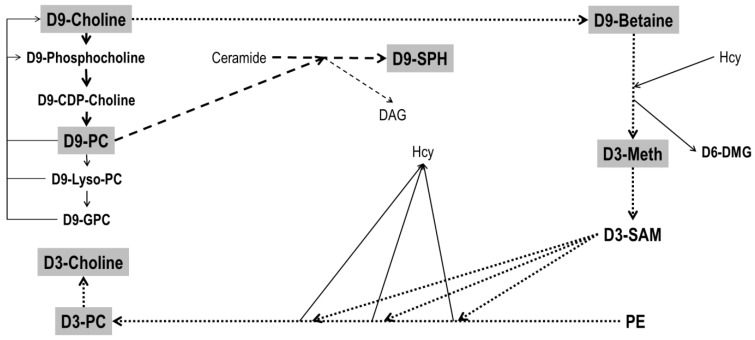
Metabolism of D9-choline. Filled fat arrows indicate de novo synthesis of D9-phosphatidylcholine (D9-PC) and its degradation products D9-Lyso-PC and D9-glycerophosphocholine (D9-GPC). D9-choline recycling is indicated by thin solid arrows. Dotted fat arrows indicate downstream metabolism of D9-choline via D9-betaine synthesis and that of D3-methionine (D3-Meth) from homocysteine. This is followed by D3-S-adenosylmethionine (D3-SAM) synthesis, serving 3-fold methylation of phosphatidylethanolamine (PE), predominantly resulting in D3-PC. The latter can undergo hydrolysis in analogy to D9-PC catabolism, resulting in free D3-choline. Striped fat arrows show the synthesis of D9-sphingomyelin (D9-SPH) from ceramide, using D9-PC as a donor of a D9-phosphocholine group. Further abbreviations: DAG, diacylglycerol; D6-DMG, D6-dimethylglycine. For further details and enzymes/micronutrients involved see ref [25].

Choline metabolism is effectively addressed by pulse-labeling with deuterated choline (D9-choline) [21,22], allowing for the assessment of its turnover and the assessment of its metabolites in vivo [23,24] (for metabolic details see: Figure 1 and [25]). Most PC is synthesized de novo, using exogenous choline via the cytidine-5’-diphosphocholine (CDP-choline) pathway [6]. SPH synthesis from ceramide by sphingomyelin synthase uses PC as a phosphocholine donor [26], and cellular PC turnover results in the formation and recycling of lyso-PC, glycerophosphocholine (GPC), phosphocholine and choline, all being D9-choline labeled under such conditions. Additionally, the liver oxidizes D9-choline to D9-betaine which is used for the synthesis of D3-labeled S-adenosyl-methionine (D3-SAM). Sequential phosphatidylethanolamine (PE) methylation by PE-N-methyltransferase predominantly results in D3- rather than D9-labeled PC (and D3-choline), discriminating exogenous from endogenous choline [22,27].

Against this background, and to evaluate the potential consequences of choline deficiency, we analyzed choline and choline metabolite pools and their metabolism, in 14 d old rats. Although these animals are not preterm, several aspects of development (neurological, alveolarization) are postnatal in rats, their circulation is similar to that of preterm infants rather than the fetus, and their growth rate of 11–15% per day is characteristic for rapid development suggesting a high choline requirement [1,28]. We investigated plasma as a carrier, and the liver, lung, cerebrum and cerebellum as the clinically most important neonatal organs, using D9-choline labeling for up to 24 h in 14 d old rats, and used sequential sampling and tandem mass spectrometry for analysis.

## 2. Materials and Methods

### 2.1. Materials

Chloroform (HPLC grade) was from Baker (Deventer, the Netherlands). Methanol, acetonitrile, water (analytical grade), choline chloride (>99%), phosphocholine, glycerophosphocholine, betaine hydrochloride and N,N-dimethylglycine were from Sigma-Aldrich (Munich, Germany). D_4_-choline (Choline-1,1,2,2-d_4_) chloride and D9-choline (N,N,N-trimethyl-d_9_) chloride were purchased from CDN Isotopes Inc (Pointe-Claire, Quebec, Canada). 1,2-diarachidoyl-sn-glycero-3-phosphocholine (PC20:0/20:0) and 1,2-dimyristoyl-sn-glycero-3-phosphocholine (PE14:0/14:0) were from Avanti Polar Lipids (Alabaster, AL, USA). All further chemicals were of analytical grade and from various commercial sources.

### 2.2. Animal Experiments

Animal experiments were approved by the local authorities and met NIH Guidelines for the Care and Use of Laboratory Animals. Female Sprague-Dawley rats were kept under standardized, specific pathogen-free conditions on a 12 h:12 h light/dark cycle, had free access to animal chow and tap water and were mated for 4 days (d) with a male rat. Litter size was adjusted to 12 within 24 h after delivery (d21-23), and pups of either sex were kept with their mothers until sacrifice at d14-15 (N = 29, 22 female). On d14, pups were weighed using an electronic scale (model 440-45; Kern & Sohn, Balingen, Germany), injected intraperitoneally (i.p.) with 50 mg/kg body weight (b.w.) D9-choline chloride (=336 nmol/g b.w.) and sacrificed after 1.5, 6 or 24 h [29]. For anesthesia, animals received 100 mg/kg b.w. ketamine together with 4 mg/kg b.w. xylazine i.p. 10 min prior to sacrifice. After opening the chest, 0.8–1.2 mL blood was aspirated into EDTA vacutainers from the right ventricle. The lungs and liver were then flushed with ice-cold isotonic saline (10–15 cm H_2_O pressure), and lung lavage fluid (LLF, ~10 mL ice-cold saline) was harvested via tracheal cannulation. Total liver, lungs without the trachea and hilus, cerebrum and cerebellum without brain stem were excised and immediately frozen in liquid nitrogen. LLF was spun at 200× *g*/4 °C for 10 min to remove cells, and EDTA blood at 1000× *g* for 10 min to harvest plasma [28,30]. Samples were kept at −80 °C until analysis.

### 2.3. Blood Plasma and Tissue Extraction

Frozen liver, lung, cerebrum and cerebellum were weighed on a SI234A analytical scale (Sartorius, Göttingen, Germany). Total plasma volume was estimated from blood volume (12.5% of body weight), hematocrit (40%) and plasma density (1.025 g/mL) [31,32]. Blood plasma (50 µL) and LLF (1 mL) were extracted according to Bligh and Byer, and tissues according to Folch et al. [33,34]. PC20:0/20:0, PE14:0/14:0 and d_4_-choline chloride were used as internal standards. After extraction and centrifugation, the lower chloroform phase was analyzed for lipids and the upper water:methanol phase for choline and its water-soluble metabolites. Extracts were stored at −80 °C and diluted to final concentration directly before analysis with LC-H-ESI-MS/MS.

### 2.4. Analysis of Choline, Water Soluble Choline Metabolites and Phospholipids

The mass spectrometry device comprised a Finnigan Surveyor Autosampler Plus, a Finnigan Surveyor MS Pump Plus, and a TSQ Quantum Discovery Ultima equipped with a heated electrospray ionization interface (H-ESI) (Thermo Fisher Scientific, Dreieich, Germany).

Choline, phosphocholine, cytidylyl diphosphocholine (CDP-choline), glycerophosphocholine, betaine, dimethylglycine (DMG), methionine, and their deuterium-labeled analogues were separated from 15 µL of a 1:7.5 (other organs) or 1:110 (liver) upper phase dilution with water, using a HILIC Plus^®^ column (2.1 × 100 mm, 3.5 μm particle size, Agilent Technologies, Böblingen, Germany) at 40 °C and an acetonitrile:water:formic acid gradient as described before [2]. Components were analyzed at positive ionization in the selected reaction monitoring (SRM) mode, using mass by charge transitions of 104→60 (choline), 107→63 (D3-choline), 108→60, 61 (D_4_-choline), 113→69 (D9-choline), 118→59 (betaine), 127→68 (D9-betaine), 184→86 (phosphocholine), 193→95 (D9-phosphocholine), 104→58 (dimethylglycine), 110→64 (D6-dimethylglycine), 258→104 (glycerophosphocholine), 267→113 (D9-glycerophosphocholine), 150→61 (methionine), 153→64 (D3-methionine), 162→60 (carnitine) and 165→63 (D3-carnitine). LLF was not investigated for water-soluble components due to the dilution of the epithelial lung lining fluid by the lavage procedure [35].

Phospholipids were analyzed from the organic phase diluted 1:157 with chloroform:methanol (60:40, vol/vol) and 25 μL injected [28,36]. Endogenous and D9-/D3-labeled PC, lyso-PC, SPH, and PE and lyso-PC were separated with a Polaris 3 Si-A column (2.0 × 100 mm; Agilent Technologies, Böblingen, Germany) at 40 °C. Chloroform:methanol:300 mM ammonium acetate (60:38:2, vol/vol) was the mobile phase, and detection was at positive ionization in the SRM mode as described before [24,28]. Phosphocholine (mass/charge [*m*/*z*] = +184) was the diagnostic fragment for endogenous PC, SPH and lyso-PC, whereas D9-phosphocholine (*m*/*z* = +193) and D3-phosphocholine (*m*/*z* = +187) served for their D9-/D3-labeled analogues. PE was analyzed via neutral loss of *m*/*z* = +141 for individual species in the SRM mode [37]. Total ion counts were corrected for the ^13^C effect and different ionization rates according to chain length. Concentrations and pools were calculated from their internal standards as described before [28]. PC and PE species were grouped according to the presence of two saturated fatty acids (sat-PC/PE), a mono-unsaturated oleic (C18:1-PC/PE), di-unsaturated linoleic (C18:2-PC/PE), arachidonic (C20:4-PC/PE) or docosahexaenoic acid (C22:6-PC/PE) residue, which accounted for >95% of whole PC or PE [14,35,36].

### 2.5. Statistics

All data were checked for normal distribution and are expressed as mean ± standard error (SE) of the indicated numbers of experiments (N). Pairs were compared by a two-tailed test, and multiple comparisons between groups were made with Tukey’s range test, and results were corrected via the method of Bonferroni, using GraphPad Instat^®^, version 3.0 (San Diego, CA, USA). Significance was accepted at *p* < 0.05.

## 3. Results

### 3.1. Pool Sizes of Unlabeled Choline and Its Water-Soluble Metabolites

The body weight of the 14 d old rats was 28.2 ± 0.4 g. Total weights of investigated organs and plasma comprised 15.6 ± 0.2% of this value, whereas their total choline-containing phospholipids amounted to 61.3 ± 3.9 µmol (Table 1), which is 21.7 ± 1.4% of the mean estimated choline-containing phospholipid pool (10 µmol/g) [1]. Detailed pool sizes of choline, its water-soluble metabolites, and phospholipids containing a choline head group are shown in Table 1A,B, indicating free choline and phosphocholine as predominant water-soluble PC precursors. Although the total choline pool was low in plasma compared to other organs, its plasma concentration was 35 ± 3µM, i.e., in the same range as in the human fetus [2]. Plasma phosphocholine and CDP-choline, however, only comprised 1.8 ± 0.3 µmol/L and 0.2 ± 0.1 µmol/L, respectively.

Free choline pools were 2–3 fold higher in the liver, cerebrum and cerebellum than in plasma and lung tissue (*p* < 0.001). Phosphocholine and CDP-choline pools for de novo PC synthesis [6] were higher in the cerebrum (2.1 ± 0.2-fold) and cerebellum (1.8 ± 0.2-fold) than in the liver, although their organ weights were identical or only 32 ± 2%, respectively, of that of the liver. These precursors were low in lung tissue (Table 1A). α-Glycerophosphocholine (GPC), the fully deacylated breakdown product of phosphatidylcholine (PC), was low in all organs (15 ± 1% relative to PC precursors) but higher in the liver compared to other organs and plasma.

The betaine pool was as high (116 ± 9%) as the sum of all PC precursors (choline, phosphorylcholine, CDP-choline), and was predominantly located in the liver and plasma over other organs (*p* < 0.001). Its demethylation product dimethylglycine (DMG), derived from methionine synthesis by betaine homocysteine methyltransferase (BHMT) [1,22], was highest in the liver and plasma as well, so that these downstream products of choline exceeded those of PC precursors (*p* < 0.05). Methionine, as well as trimethylamine oxide (TMAO) derived from bacterial degradation of choline to trimethylamine (TMA), followed by its hepatic oxidation [38], were low compared to other components. Interestingly, TMAO was enriched in plasma and lung tissue (*p* < 0.001) (Table 1A).

### 3.2. Pool Sizes of Unlabeled Choline-Containing Phospholipids

Phospholipids containing a choline head group were dominated by PC, followed by SPH and lyso-PC. Their concentrations were 2.6 ± 0.1 µmol/mL in plasma, and ~10 fold higher in the liver, lung, cerebrum and cerebellum (28.0 ± 1.4, 23.6 ± 1.2, 21.3 ± 1.7 and 21.5 ± 0.9 µmol/g, respectively). Consequently, due to their respective organ weights, the brain contained as much PC, SPH and lyso-PC as the liver (together 70 ± 4% of investigated organs’ pool size). Plasma, the lung and cerebellum contained 9.0 ± 0.3%, 10.8 ± 0.5% and 9.4 ± 1.0%, respectively, of the total, whereas lung lavage fluid (LLF) representing surfactant, only comprised 0.8 ± 0.1% of choline-phospholipids (Table 1B). Notably, although SPH was a minor fraction of phospholipids in all organs, lung tissue comprised 2.9 ± 0.1 fold more SPH than the cerebellum (*p* < 0.001) in spite of their similar organ weight and PC content (Table 1B).

In essence, choline-phospholipid pools are dominated by similarly high PC concentrations in parenchymal organs, with pool sizes—and choline requirements—according to organ weight. Pools were comparably high in the liver and cerebrum, but smaller in the lung, cerebellum and plasma. Among all choline metabolites, betaine was the second most abundant, exceeding the pools of other water-soluble components.

### 3.3. Kinetics of D9-Choline and Its Water-Soluble Deuterated Metabolites

**Figure 2:**
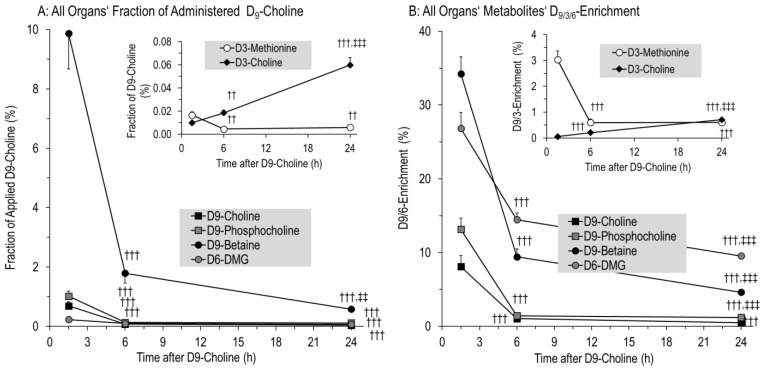
Fraction of administered D9-choline (**A**) and deuterium enrichment (**B**) in water-soluble components of the sum of all investigated organs. Fractions of administered D9-choline (**A**) were corrected for the number of deuterated methyl groups in the respective components, which are 2 for D6-dimethylglycine (D6-DMG), and 1 for D3-methionine and D3-choline. Data are means ± SE of 8–10 data points per time point (1.5 h, 6 h, 24 h). Abbreviations: ††, *p* < 0.01, †††, *p* < 0.001 vs. 1.5 h; ‡‡, *p* < 0.01, ‡‡‡, *p* < 0.001 vs. 6 h.

According to their body weight and dosage (50 mg/kg), rat pups received 9456 ± 146 µmol D9-choline. The fractions of water-soluble and lipidic D9-choline metabolites and derivatives are indicated in Table 2. At 1.5 h, 11.8 ± 1.5% of applied D9-choline was found as water-soluble metabolites for the sum of studied organs and plasma. Of these, 9.9 ± 1.2% was D9-betaine, whereas total betaine comprised only 3.0 ± 0.2% of the total choline (metabolite) pool. This was followed by D9-phosphocholine, D9-choline, D6-DMG and D9-GPC (Figure 2A). At 6 h and 24 h, water-soluble D9-choline metabolites were nearly absent, having decreased by 7.3- and 15.6-fold (2.10 ± 0.31% and 0.98 ± 0.09% of applied D9-choline label, respectively) (*p* < 0.001).

**Figure 3:**
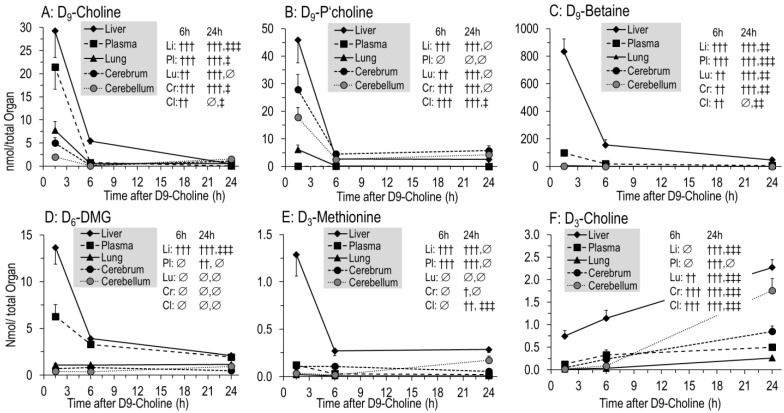
Kinetics of D9-choline (**A**) and its deuterated water-soluble metabolites (**B**–**F**) in individual organs. Data are expressed as total pools of compounds in individual organs and total plasma volume and are means ± SE of 8–10 data points per time point (1.5 h, 6 h, 24 h) after intraperitoneal injection of 50 mg/kg D9-choline chloride. Abbreviations: ∅, not significant; †, *p* < 0.05, ††, *p* < 0.01, †††, *p* < 0.001 vs. 1.5 h; ‡, *p* < 0.05, ‡‡, *p* < 0.01, ‡‡‡, *p* < 0.001 vs. 6 h.

Detailed kinetics of water-soluble D9-choline-containing or -derived components are shown in Figure 3 for individual organs, demonstrating an overall rapid decrease in D9-choline, D9-phosphocholine, D9-betaine, D6-DMG and D3-methionine (all *p* < 0.001) (Figure 3A–E). Initial hepatic D9-labeled pools of water-soluble components surmounted those of other organs (Figure 3A–F), followed by plasma for D9-choline, D9-betaine and D6-dimethylglycine (D6-DMG). D9-phosphocholine as an intracellular PC precursor was absent from plasma. While D9-choline was lower in the cerebrum and cerebellum than in lung tissue, D9-phosphocholine in these organs was higher at 1.5 h (*p* < 0.001). Contrary to the liver and plasma, D9-betaine, D6-DMG and D3-methionine were generally low in the lung and brain (Figure 3C–E).

Notably, D9-PC precursors were nearly absent from tissues at 6 h to 24 h (Figure 2A,B), indicating no significant D9-PC synthesis in tissues from 6 h onwards, contrasting the severalfold increase in D9-choline labeled PC in the lung and brain from 6–24 h (see below).

In contrast to these kinetics, pools and enrichment of D3-choline, derived from D3-PC synthesis by phosphatidylethanolamine (PE) methylation and requiring D9-betaine for D3-SAM formation and PE-N-methyltransferase (PEMT) [22,24], continuously increased (R^2^ = 0.9991; *p* < 0.001). However, D3-choline comprised less than 0.1% of administered D9-choline (Figure 2A,B inserts).

### 3.4. Kinetics of Phospholipids Containing D9- or D3-Choline

**Figure 4:**
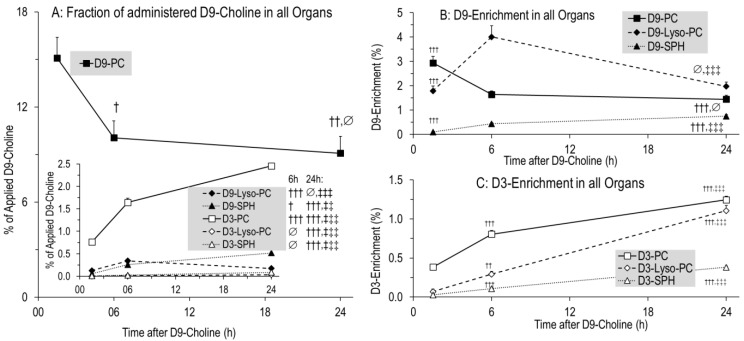
Fractions of administered D9-choline (**A**) and enrichment of D9-choline (**B**) and D3-choline (**C**) labeled phospholipids in the sum of all investigated organs (liver, lung, lung lavage fluid, cerebrum, cerebellum) and total plasma. For fractions of D3-labeled components (**A**), absolute values were divided by 3 as only one of 3 D3-methyl groups of the administered D9-choline are present in the target molecules. Data are means ± SE of 8–10 experiments and indicate the kinetics from 1.5 h to 24 h after 50 mg/kg intraperitoneal D9-choline chloride injection. Abbreviations: D9/3-PC, D_9/3_- phosphatidylcholine; D_9/3_-Lyso-PC, D_9/3_-lyso-phosphatidylcholine; D_9/3_-SPH, D_9/3_-sphingomyelin. ∅, not significant; †, *p* < 0.05, ††, *p* < 0.01, †††, *p* < 0.001 vs. 1.5 h; ‡‡, *p* < 0.01, ‡‡‡, *p* < 0.001 vs. 6 h.

Deuterated phospholipids (Figure 4) were characterized by those containing the original tracer (D9-PC, D9-lyso-PC, D9-SPH), or a single D3-methyl group (D3-PC, D3-lyso-PC, D3-SPH) derived from PE methylation (see above) [22,24]. After 1.5 h, 15.3 ± 1.4% of the applied tracer was present in D9-labeled phospholipids of organs and plasma, mainly as D9-PC (15.1 ± 1.3%) (Figure 4A). In contrast to water-soluble metabolites, pools of these D9-labeled phospholipids only decreased to 10.6 ± 1.1% of the injected label at 6 h, and then remained constant (9.7 ± 1.1% at 24 h, *p* > 0.05) (Figure 4A). By contrast, D3-PC continuously increased, but its fraction comprised only 2.5 ± 0.1% of administered D9-choline at 24 h (Figure 4A, insert). Accordingly, the D3-enrichment of phospholipids was lower compared to that of their D9-labeled counterparts (Figure 4C). Other minor deuterated phospholipids (<1% of the initial label) showed either a continuous (D9-SPH, D3-Lyso-PC; all *p* < 0.01) or intermittent (D9-Lyso-PC; max = 6 h; *p* < 0.01) increase (Figure 4A, insert).

### 3.5. Organ-Specific Kinetics of Phospholipids Containing D9- or D3-Choline

**Figure 5:**
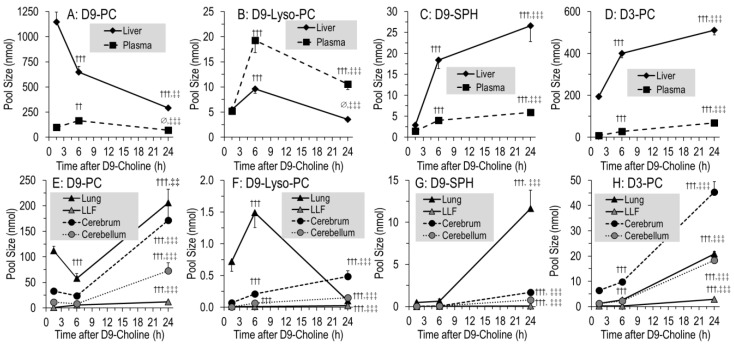
Pool size kinetics of D9-PC (**A**,**E**), D9-lyso-PC (**B**,**F**), D9-SPH (**C**,**G**) and D3-PC (**D**,**H**) in different organs. Data are means ± SE of 8–10 data points at 1.5 h, 6 h and 24 h, respectively, after intraperitoneal administration of 50 mg/kg D9-choline chloride. Abbreviations: D_9/3_-PC, D_9/3_-phosphatidylcholine; D9-Lyso-PC, D9-lyso-phosphatidylcholine; D9-SPH, D9-sphingomyelin, LLF, lung lavage fluid; ∅, not significant, ††, *p* < 0.01, †††, *p* < 0.001 vs. 1.5 h; ‡‡, *p* < 0.01, ‡‡‡, *p* < 0.001 vs. 6 h.

Figure 5 shows the pool changes of deuterated phospholipids in the liver and plasma (Figure 5A–D) compared to the lung, LLF, cerebrum and cerebellum (Figure 5E–H). It shows the domination of D9-PC (Figure 5A,E) over all other deuterated compounds (Figure 5B-D,F–H), and of the liver over all other organs. In the liver, D9-PC decreased from 1145 ± 98 nmol at 1.5 h to 647 ± 58 nmol at 6 h (−43%) (Figure 5A). Similarly, lung tissue D9-PC decreased (112 ± 9 to 58 ± 9 nmol, −48%), whereas no decrease occurred in the cerebrum and cerebellum (Figure 5E). D9-PC of surfactant (LLF), as a separate compartment with high intra-alveolar recycling, continuously increased but was of a small amount (0.4 ± 0.1 to 5.7 ± 0.5 nmol) (Figure 5E). Pools of D9-Lyso-PC, D9-SPH and D3-PC had different kinetics, but accounted only for minor fractions of applied D9-choline, indicating that choline metabolism is ruled by D9-PC.

While total D9-PC was constant from 6 h onwards (Figure 4A), its pulmonary, cerebral and cerebellar values increased by 143 ± 21 nmol, 148 ± 22 nmol and 65 ± 14 nmol, respectively, at the expense of the liver (*p* < 0.001) (Figure 5A,E). This occurred at a time when D9-labeled PC precursors were virtually absent from tissues (see above). At the time of low D9-PC levels in the liver and lung (6 h), it was highest in plasma but decreased thereafter (Figure 5A).

Different kinetics were seen for minor components, such as D9-lyso-PC (Figure 5B,F) and D9-SPH requiring preexisting D9-PC (Figure 5C,G), or D3-PC (Figure 5D,H) requiring D9-betaine/D3-methionine for synthesis (Figure 1), partly increasing throughout. Notably, D9-SPH synthesis was several-fold higher in the lung compared to the cerebrum and cerebellum (*p* < 0.001) (Figure 5G), which is in line with its high fraction among endogenous lung tissue phospholipids (Table 1B).

### 3.6. Kinetics of Newly Synthesized PC Sub-Groups

**Figure 6:**
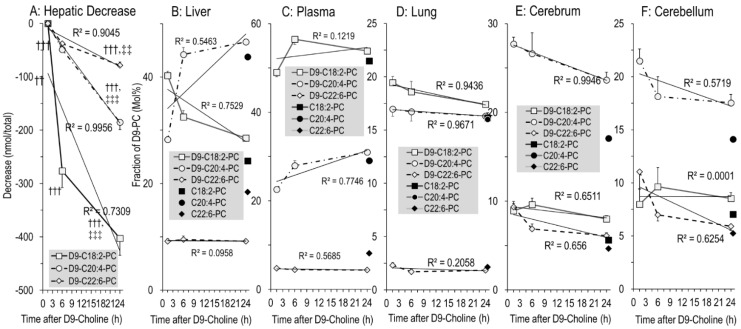
The absolute decrease in poly-unsaturated D9-labeled PC subgroups in the liver (**A**), and their fractions in D9-labeled versus endogenous PC in the liver (**B**), plasma (**C**), lung (**D**), cerebrum (**E**) and cerebellum (**F**). C18:2-PC (■), C20:4-PC (●) and C22:6-PC (♦) represent the endogenous PC sub-groups containing a linoleic (C18:2n-6), arachidonic (C20:4n-6) or docosahexaenoic (C22:6n-3) acid residue, respectively, of all time points (N = 27), whereas their D9-choline-labeled analogues are indicated by open symbols (D9-C18:2-PC [□], D9-C20:4-PC [○] and D9-C22:6-PC [◊], respectively). Data are means ± SE at the respective time points (N = 8–10). †††, *p* < 0.001 vs. 1.5 h; ‡‡, *p* < 0.01, ‡‡‡, *p* < 0.001 vs. 6 h.

Fatty acid composition of hepatic PC secreted into plasma is characteristically different from that of total liver PC [22], and different in other tissues as well [21,39,40]. Therefore, we investigated the kinetics of polyunsaturated D9-PC subgroups of tissues relative to equilibrium composition (unlabeled PC). In the liver, the amounts of newly synthesized D9-PC containing a linoleic acid (LA) residue (D9-C18:2-PC) decreased faster than those containing arachidonic (ARA, C20:4) (D9-C20:4-PC) or docosahexaenoic acid (DHA, C22:6) (D9-C22:6-PC) (Figure 6A). Hence, fractions of D9-C20:4-PC and D9-C22:6-PC increased with time, reaching values close to equilibrium PC composition (Figure 6B). In line with this, D9-C18:2-PC was higher compared to D9-C20:4-PC and D9-C22:6-PC in plasma than in the liver (all *p* < 0.001, Figure 6C), suggesting preferential hepatic secretion of PC containing an LA residue. For other organs (Figure 6D–F), the composition of D9-labeled PC sub-groups was either identical to organotypic unlabeled PC throughout (lung, Figure 6D) or approached equilibrium within 24 h (cerebrum and cerebellum, Figure 6E,F), suggesting selective uptake or local fatty acid modulation. This similarly applied to D3-PC (supplementary Figure S1).

Notably, high enrichment in C20:4-PC compared to C22:6-PC (16.0 ± 0.1% vs. 2.1 ± 0.1% (Figure 6D) was characteristic for the lung, whereas in the cerebrum and cerebellum C20:4-PC predominated (17.1 ± 0.2% and 14.1 ± 0.1%, respectively) over both C18:2-PC (6–7%) and C22:6-PC (4.7 ± 0.1% and 5.2 ± 0.1%, respectively (Figure 6E,F)). Analysis of PE, the second major tissue phospholipid, showed an even higher enrichment in C20:4 and C22:6 compared to PC, indicating high pools and abundance of these poly-unsaturated fatty acids in tissues (supplementary Figure S2).

## 4. Discussion

Choline is a rapidly metabolized essential nutrient, with its concentration and those of its metabolites being tightly regulated in plasma and tissues. Concentrations of PC and SPH, comprising 85–90% of the total choline pool, are highest in parenchymal organs, liver, lung and brain. They serve membrane structure, fat emulsion (bile) and reduction of surface tension (surfactant), as well as the synthesis of signaling molecules. In the form of PC, choline is essential for the transport of arachidonic (ARA, C20:4) and docosahexaenoic (DHA, C22:6) acid in plasma lipoproteins, whereas membrane PC serves as a reservoir of these fatty acids to be released for eicosanoid and docosanoid synthesis. Moreover, PC serves as the synthesis/regeneration of SPH from ceramides that contribute to apoptosis [17,41]. Finally, via its oxidation product betaine, choline feeds the one-carbon pool as a methyl donor, for S-adenosylmethionine (SAM) synthesis as the ubiquitous “currency” of methylation processes. Hence, choline is central to homeostasis, growth and organ function in health and disease. The untimely postnatal decrease in plasma choline concentration observed in preterm infants, and the apparently insufficient supply and resulting cumulating choline deficit of these patients [1,2,5,24,42,43] led us to investigate in neonatal rats the pools, metabolism and trafficking of choline and its derivatives in the liver, lung and brain, the most critical organs of preterm infants. Although 14 d old rats are not preterm, and do not undergo the plethora of stress factors of preterm infants on a neonatal intensive care unit (NICU), neonatal rats are characterized by several similarities to preterm infants: their circulatory and respiratory physiology are similar, both are born very immature with respect to postnatal alveolarization of the lungs and brain development, and, finally, the physiologic growth rate of 14 d old rats is extremely high, resembling that of humans at 24–34 wk postmenstrual age (PMA) rather than that of term infants (>37 wk PMA) [1,27]. Hence, the authors suggest that neonatal rats are a useful model to investigate the metabolism of choline with respect to preterm infants.

### 4.1. Overall Concentrations and Pools of Choline and Choline Metabolites in Targeted Organs

To optimally address quantitative aspects of choline metabolism, we used animals with adjusted litter size (N = 12) to assure comparable growth, and maternal milk feeding for a supposedly adequate nutrient supply, including choline and other nutrients.

Plasma choline in the rapidly growing neonatal rat equaled human fetal plasma concentrations (35 ± 3µM) [2], to ensure rapid cellular choline uptake for PC synthesis and parenchymal growth. Importantly, we found no sex differences of plasma choline in human cord blood before 37 wk PMA, and later on, lower values of female fetuses fairly exceeded those of preterm infants and their mothers, suggesting that sex differences are of minor relevance here [2]. Such high plasma choline concentration during rapid growth corresponds to the high K_M_ values of ubiquitous choline transporters, allowing for cellular choline uptake proportional to concentration [6,7]. This contrasts with the rapid untimely postnatal decrease in preterm infants’ plasma choline concentration [2]. Concentrations of PC and SPH in tissues and plasma equaled those of adult organisms (21–28 µmol/g and 2.55 ± 0.01 µmol/mL, respectively) [6,44], indicating that they are kept constant during development and increase proportionally to parenchymal growth. Consequently, a cumulative deficit due to low supply will necessarily impact parenchymal growth that is generally impaired in preterm infants [1,6,17].

The total of investigated tissues and plasma comprised 15.7 ± 0.4% of animal weight (see Table 1). By contrast, 61.3 ± 3.9 µmol choline-containing phospholipids equal 21.7 ± 1.4% of estimated total pool size (282 ± 4 µmol at estimated 10 µmol/g b.w. [1]), indicating choline enrichment in the target organs of this study: Similar to the human fetus [45], the liver comprised 2.9 ± 0.1% of body mass, but 8.1 ± 0.4% of total choline-lipids, pointing to the central role of the liver in choline metabolism. The cerebrum comprised 3.1 ± 0.1% of body mass, but 7.1 ± 0.6% of choline lipids, and cerebellar values were 1.0 ± 0.05% and 2.1 ± 0.2%, respectively, highlighting the quantitative aspects of choline requirements for brain development. Similarly, neonatal rat lungs comprised 1.00 ± 0.01% of body mass, but 2.4 ± 0.1% of the body’s choline-containing phospholipids. This is particularly important, as the liver is prioritized over other organs during choline deficiency [10], and the lung is used to supply the liver during choline deficiency [8]. Notably, as pulmonary choline/PC&SPH pools are much lower than those of the liver, lung tissue may easily become choline-deprived, if it is used to feed the liver with choline (via HDL) during deficiency [8,15].

### 4.2. Overall Kinetics of D9-Choline, Its Water-Soluble Metabolites and D9-PC

Usually, choline is recognized as a precursor for PC and SPH synthesis for membranes, lipoproteins, bile and surfactant, and for the synthesis of acetylcholine (ACh) for neurotransmission. However, the large pools of betaine and DMG as downstream products of choline, particularly in the liver and plasma, (Table 1) surmount those of PC precursors. Approximately 10% of applied D9-choline were found in D9-betaine at 1.5 h, compared to ~15% in phospholipids, showing that major proportions of administered D9-choline are oxidized to betaine. Moreover, such initial synthesis exceeded the fraction of endogenous betaine (~3% of total choline metabolites) by 3-fold, and D9-betaine values rapidly decreased to 1.8 ± 0.3% and 0.6 ± 0.1% at 6 and 24 h, respectively, highlighting rapid betaine turnover and its consumption as a methyl donor in the immature organism. In spite of the central role of the liver in choline and betaine metabolism, the kidneys, comprising 20–25% of liver weight, contribute to betaine formation as well [9]. In line with this and the similarly high betaine formation in preterm infants as indicated by D9-betaine formation from D9-choline [24], data point to the necessity of including betaine formation in the estimation of choline requirements in preterm infant nutrition.

Beyond this, betaine was found in plasma, followed by other tissues (see Figure 3), which is in line with its function in osmotic regulation and lung protection from acute and chronic injury [46,47,48]. By contrast, synthesis of D3-PC and D3-choline, requiring D9-betaine as a methyl donor via D3-methionine/D3-SAM (PEMT pathway) [26]) was low, confirming that betaine is not primarily used for endogenous choline synthesis in neonatal rats as in preterm infants [24].

### 4.3. Overall and Hepatic Kinetics of D9-PC Precursors for De Novo PC Synthesis

At 1.5 h, D9-choline was low, but still present in plasma. Less than 2% of the administered tracer was present as D9-PC precursors (D9-phosphocholine and D9-CDP-choline) and <0.2% at 6–24 h. Although we did not follow D9-choline kinetics prior to 1.5 h, data indicate high initial plasma and organ concentrations, and rapid plasma and tissue turnover of choline [21,40]. Such a rapid disappearance of deuterated precursors is in line with the high concentration of D9-labeled phospholipids, pointing to a rapid and complete precursor consumption for de novo synthesis of D9-PC in all organs [21,40,49], surmounting precursor concentrations at 1.5 h by far. Such rapid D9-choline turnover further suggests that any choline supplementation in preterm infants has to be continuous to achieve persistently high plasma concentrations to meet physiological requirements [1,24,40]. Moreover, the absence of D9-labeled PC precursors from 6 h onwards, together with low concentrations of D9-PC degradation products, such as D9-GPC and D9-lyso-PC, indicates that the several-fold increase in D9-labeled PC in organs beyond 6 h must primarily come from inter-organ exchange via plasma, where D9-PC and other D9-labeled phospholipids, but not their deuterated precursors were detected.

### 4.4. 1.5–6 h Kinetics of D9-PC in the Lung Compared to the Liver

Data show a decrease in both hepatic and pulmonary D9-PC stores from 1.5 to 6 h after D9-choline spiking, and a corresponding increase in plasma D9-PC (Figure 5A,E). As no (D9-choline, D9-phosphocholine) or very low (D9-GPC, D9-lyso-PC) concentrations of D9-PC degradation products were found beyond 1.5 h, hepatic and pulmonary decreases indicate secretion into plasma. In plasma, PC is located in very-low-density lipoproteins (VLDL, ~20% PC) from the liver, but also in HDL (~20% PC) originating from pulmonary (and other organs’) basolateral PC secretion via ABC-A1 transporters [16,50,51]. Whereas we did not differentiate plasma lipoproteins, our data are consistent with the increasing concentration in plasma D9-PC from 1.5 to 6 h due to other organs’ secretion. Notably, similar to preterm infants, values of plasma D9-PC in neonatal rats were highest at 6 h compared to 24 h in adults, demonstrating accelerated choline/PC turnover in neonatal organisms [22,24,40].

Although hepatic PC is secreted into the intestinal lumen via bile as well [52], biliary and intestinal D9-PC metabolism were not addressed in this study. However, the presence of D9-trimethylamine oxide (D9-TMAO), resulting from bacterial D9-choline/PC degradation in the intestinal lumen [37], shows a contribution of the intestine to D9-choline/PC metabolism after intraperitoneal application.

While liver and plasma D9-PC kinetics are explained by hepatic PC/VLDL secretion, decreased D9-PC pools at 6 h were similarly found in lavaged lung tissue. This decrease (−54 ± 9 nmol) is not explained by intrapulmonary catabolism, as its degradation product lyso-D9-PC was low, and water-soluble metabolites (D9-GPC, D9-phosphocholine, D9-choline) were virtually absent. Similarly, surfactant secretion resulted in an increase in D9-PC in LLF of only 5.3 ± 0.5 nmol from 1.5 to 6 h (Figure 4E). Hence, the only remaining explanation of the D9-PC decrease in lung tissue is its secretion into the circulation. This was described for adult lungs before, where the major fraction of newly synthesized PC is basolaterally secreted by adenosine triphosphate binding cassette (ABC) transporter A1 (ABC-A1) of pneumocytes type II and transferred to apo-lipoprotein A1, thereby contributing to systemic choline homeostasis via HDL [8,15,16]. Our data suggest pulmonary PC secretion into the circulation in the neonatal organism as well [15,28].

In essence, increased plasma D9-PC at 6 h may reflect both hepatic VLDL and pulmonary HDL secretion (Figure 5). Further studies are essential to directly quantify the lungs’ and other organs’ contribution to systemic choline/PC homeostasis. Notably, pulmonary choline/PC secretion and its hepatic accretion during choline deficiency [8] may impair lung development, particularly because pulmonary mass and choline/PC pools are only 25–30% of the hepatic ones (see Table 1 and [1,45]), pulmonary choline/PC secretion may easily result in choline exhaustion of the growing lung during choline deficiency.

### 4.5. 1.5–6 h Kinetics of D9-PC in LLF

In contrast to lung tissue, LLF representing surfactant showed a continuous increase in D9-PC. Similar to the liver, apical surfactant secretion is separated from basolateral PC secretion contributing to systemic lipid homeostasis (see above). This is analogous to the separation of apical hepatic PC secretion into bile (and its enterohepatic cycle) from the liver’s contribution to systemic PC metabolism [53]. However, whereas apical secretion of the liver into bile consumes at least 50% of its PC pool per day, surfactant secretion consumes only a minor fraction of pulmonary PC synthesis [28,51], suggesting that choline requirements of the lungs are dominated by its parenchymal requirement and contribution to systemic lipid metabolism.

### 4.6. 1.5–6 h Kinetics of D9-PC in the Cerebrum and Cerebellum

In contrast to the liver and lung, no significant decrease in D9-PC was observed in the cerebrum and cerebellum from 1.5 h to 6 h. This indicates that the brain does not significantly contribute to systemic choline/PC trafficking and homeostasis. Nevertheless, our data and other studies [4,9] indicate that the developing brain depends on exogenous choline supply. According to the total organ and choline pool sizes of the cerebrum and cerebellum, the quantitative requirements for brain development exceed those of the liver and lung.

### 4.7. 6–24 h Kinetics: D9-PC Accretion in the Lung and Brain

The constant total amount of D9-PC and other D9-choline labeled phospholipids from 6–24 h, in the sum of investigated organs, contrasts their changes in individual organs. Whereas D9-PC further decreased from 6 h to 24 h in the liver (and plasma), it increased in the lung, cerebrum and cerebellum. During this time, water-soluble D9-labeled PC precursors were virtually absent from these organs and plasma, suggesting direct D9-PC accretion by lung and brain at the expense of the liver via plasma. Consequently, choline/PC homeostasis of the liver is critical to the supply of other organs in the form of PC (Figure 5). The low amounts of D9-PC found in plasma compared to the liver and other organs are in line with rapid accretion of plasma D9-PC by the lung and brain, showing steep D9-PC increases. Hence, the liver significantly contributes to choline/PC availability in these organs, and therefore to their growth. This central role of the liver may be impaired by nutritional choline deficiency in preterm infants, where hepatic choline/PC supply of the lung and brain may be diminished. However, this will have to be quantified in further experiments using choline-deprived neonatal rats.

### 4.8. Organotypic Composition and Metabolism of D9-PC Subgroups

PC has unique organotypic fatty acid compositions that differ between organs and secretions and during development [21,22,24,28,35,38,52]. Whereas surfactant is enriched in disaturated PC species, fetal plasma comprises high concentrations of PC containing ARA or DHA (C20:4-PC, C22:6-PC), besides PC containing a LA or oleic acid residue (C18:2-PC, C18:1-PC). In adults, C18:1-PC and C18:2-PC are synthesized de novo, but C22:6-PC and C20:4-PC are primarily synthesized by hepatic PE methylation (PEMT pathway) [22]. However, in the fetus and preterm infant, the large amounts of C20:4-PC and C22:6-PC are derived from de novo synthesis requiring exogenous choline and high ARA/DHA relative to LA supply, as the PEMT pathway is nearly absent [24,35,54].

Importantly, C18:2-PC was preferentially secreted into plasma by the neonatal liver, as D9-C18:2-PC decreased much faster than D9-C20:4-and D9-C22:6-PC, and was high in plasma over hepatic values (see Results, Figure 6). This contrasts to the human fetus, where C20:4-PC is high at the expense of C18:2-PC throughout gestation, and C22:6-PC increases beyond 32 w gestational age [35]. Moreover, this (physiologic) fetal situation rapidly reverses after birth, due to nutrition with high amounts of LA [17]. The preferential hepatic secretion of LA over ARA and DHA via PC observed herein is in line with the postnatal changes in preterm infants where such changes occur at an unphysiologically early stage of development, due to alimentary fatty acid mismatch [35,55].

Both PC at equilibrium (unlabeled) and newly synthesized (D9-labeled) poly-unsaturated PC sub-groups (C18:2-PC, C20:4-PC, C22:6-PC) were different in the lung and brain compared to liver and plasma, and their D9-label showed their preferential de novo synthesis. Local synthesis of D9-PC beyond 6 h was excluded by the absence of D9-labeled PC precursors. Hence, the approximation of D9-PC composition to an organotypic profile within 24 h, must be due to selective PC uptake or fatty acyl remodeling (Lands Cycle) that is not detectable by D9-choline labeling.

Further studies will be necessary to address the activity of Land’s cycle in the neonatal brain and lung. Principally, PLaseA2 cleaves the sn-2-fatty acid from PC, followed by re-acylation by organotypic lyso-PC acyltransferase 1 (LPCAT1) in the lung, and LPCAT2 and 4, favoring ARA (C20:4) and DHA (C22:6), in the brain. Notably, such remodeling requires the availability of ARA and DHA, which are enriched in PE (supplementary Figure S2), the second major tissue phospholipid. However, in preterm infants ARA and DHA tissue stores are depleted in both PC and PE, resulting in a cumulative deficit similar to that of choline due to insufficient supply [1,17,35,56].

### 4.9. D9-Betaine Metabolism, Synthesis of Endogenous D3-PC, D3-Choline and D9-SPH

The metabolic consumption of D9-betaine for D3-SAM synthesis is confirmed by the presence of D6-dimethylglycine (D6-DMG), D3-methionine, D3-PC derived from the PEMT pathway and free D3-choline [22]. However, total amounts of D3-PC and D3-choline were low compared to D9-PC and D9-betaine, suggesting low activity of this pathway in neonatal rats, as also found in choline-deficient preterm infants [24,53]. Nevertheless, such low PEMT activity still contributes to DHA-PC formation in choline-supplemented preterm infants [24]. Notably, our data show that the PEMT pathway and endogenous choline synthesis are linked to choline supply, as the D3-label is derived from exogenous D9-choline. D3-PC, D3-choline and D9-SPH showed a continuous increase, indicating that all components derived from D9-choline metabolites (D9-betaine, D9-PC) are continuously released from primarily synthesized choline metabolites. For SPH, this may be particularly important to the lung. Here, SPH was increased 3-fold compared to PC and showed a higher D9-SPH synthesis rate compared to other organs. As SPH synthesis from ceramides requires PC as a phosphocholine donor, and as pro-apoptotic ceramides are increased in injured lungs, choline deficiency may further impair lung tissue recovery via decreased availability for ceramide/sphingolipid metabolism [57,58,59,60].

## 5. Conclusions

**Figure 7:**
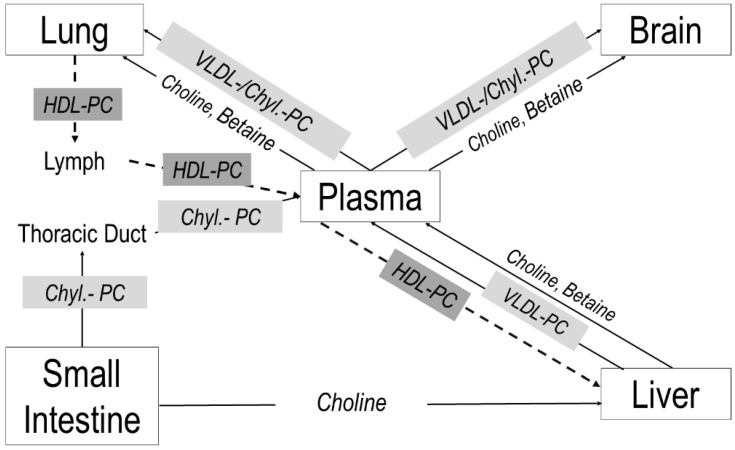
Choline trafficking in neonatal target organs. Arrows indicate the flux directions of choline and its metabolites between the lung, brain, intestine and liver via plasma and lymph fluid. Abbreviations: Chyl, chylomicron; PC, phosphatidylcholine; HDL, high-density lipoproteins, VLDL, very-low-density lipoproteins.

An overview of systemic choline metabolism is shown in Figure 7. The neonatal liver synthesizes large amounts of PC and betaine and distributes these compounds together with free choline via plasma to feed the lung and brain (Figure 7, solid arrows). Uptake of hepatic PC by the lung, cerebrum and cerebellum represents a significant component of their choline/PC supply. Our data point to the central role of a sufficient choline availability of the liver, to maintain peripheral and central nervous system supply, although the intestine contributes to choline homeostasis, via chylomicrons (8% PC) and delivery of choline to the liver as well ([9]; not addressed in this study). The lung, however, also releases PC into the circulation, contributing to HDL metabolism (Figure 7, dashed arrows), which in choline deficiency may lead to pulmonary PC deprivation (potentially contributing to altered lung development) to maintain liver function.

In the lung and brain, exogenous (D9-)PC rapidly assimilates to its organotypic molecular fatty acid profile, requiring an adequately high co-supplementation of choline, ARA and DHA. Choline deprivation is critical to the lung, as its PC availability is essential for parenchymal growth as well as for the regulation of ceramide metabolism and, potentially, anti-apoptosis via SPH synthesis from ceramides.

Finally, betaine synthesis for essential methylation processes accounts for at least 40% of choline consumption, suggesting its inclusion in the estimation of choline requirements, although its use for endogenous choline synthesis is of minor importance in preterm infants. These metabolic mechanisms explain why choline deficiency in preterm infants likely impairs function and development of the liver, brain and lung.

## 6. Patents

The Tübingen University Hospital, 72076 Tübingen, Baden-Wuerttemberg, Germany, is the owner of a study-related patent filing named CHOLINARA-BPD (Germany 10 2015 101 273.1; Europe: 16 703 742.3; US-Patent 10, 898,454).

## Figures and Tables

**Table 1:** Pool Sizes of Endogenous (Unlabeled) Choline, Choline Metabolites and Choline-containing phospholipids.

(A)											
Water-Soluble	Organ Weight	Choline	Phospho-choline	CDP-Choline	All PC Precursors	GPC	Betaine	DMG	Choline Derivatives	TMAO	Methionine
Organ	mg	Pool Size (nmol)									
Liver	831 ± 21	194 ± 13	98 ± 8	1.0 ± 0.2	293 ± 19	109 ± 8	1631 ± 125	39 ± 2	1657 ± 125	1.0 ± 0.3	25 ± 3
Plasma ^1^	2151 ± 26	75 ± 7 ^†††^	3.8 ± 0.6 ^†††^	0.4 ± 0.1	79 ± 7 ^†††^	9 ± 1 ^†††^	242 ± 22 ^†††^	16 ± 1	252 ± 23 ^†††^	3.0 ± 0.8 ^†††^	4.4 ± 0.3 ^†††^
Lung	282 ± 4	92 ± 12 ^†††^	29 ± 2 ^†††^	0.2 ± 0.03	122 ± 13 ^†††^	34 ± 2 ^†††^	29 ± 2 ^†††, ‡‡‡^	2.6 ± 0.2	30 ± 2 ^†††, ‡‡‡^	1.6 ± 0.4 ^†††^	3.8 ± 0.5 ^†††^
Cerebrum	881 ± 37	230 ± 17	375 ± 31 ^†††^	16 ± 2	621 ± 45 ^†††^	55 ± 13 ^†††^	21 ± 2 ^†††, ‡‡‡^	7.7 ± 0.6	26 ± 2 ^†††, ‡‡‡^	0.31 ± 0.07	24.6 ± 4.1
Cerebellum	268 ± 14	193 ± 29	329 ± 36 ^†††^	13 ± 1	533 ± 63 ^†††^	36 ± 7 ^†††^	15 ± 2 ^†††, ‡‡‡^	6.5 ± 0.9	20 ± 2 ^†††, ‡‡‡^	0.22 ± 0.03	19.2 ± 4.9
Total	4411 ± 102	785 ± 36	834 ± 43	30 ± 2	1647 ± 68	243 ± 16	1939 ± 142	72 ± 3	1986 ± 143 ^#^	6.2 ± 1.4	77 ± 7
(**B**)											
B: Lipids	Organ Weight	PC			Lyso-PC		SPH		Choline Phospholipids		
Organ	mg	Pool Size (µmol)									
Liver	831 ± 21	19.9 ± 1.0			0.19 ± 0.010		2.71 ± 0.10		22.8 ± 1.0		
Plasma ^1^	2151 ± 26	4.7 ± 0.2 ^†††^			0.44 ± 0.03 ^†††^		0.39 ± 0.02 ^†††^		5.5 ± 0.2 ^†††^		
Lung	282 ± 4	5.6 ± 0.3 ^†††^			0.052 ± 0.004 ^†††, ‡‡‡^		1.0 ± 0.1 ^†††, ‡‡‡^		6.7 ± 0.3 ^†††^		
LLF	-	0.50 ± 0.04 ^†††^			0.002 ± 0.001 ^†††^		0.01 ± 0.00 ^†††, ‡‡‡^		0.51 ± 0.05 ^†††^		
Cerebrum	881 ± 37	19.0 ± 1.6			0.063 ± 0.007 ^†††, ‡‡‡^		0.87 ± 0.09 ^†††, ‡‡‡^		20.0 ± 1.701		
Cerebellum	268 ± 14	5.4 ± 0.6 ^†††^			0.019 ± 0.003 ^†††, ‡‡‡^		0.35 ± 0.03 ^†††^		5.8 ± 0.601 ^†††^		
Total	4411 ± 102	55.2 ± 3.7			0.765 ± 0.049		5.3 ± 0.3		61.3 ± 3.9		

Tissues were extracted as described in Materials and Methods. Choline, its water-soluble metabolites, PC, lyso-PC and SPH were then analyzed with LC-MS/MS. Data are pool sizes as the sum of endogenous and deuterated (D_9_/D_3_) compounds and are indicated as means ± SE of 27 animals. ^1^, Applies to plasma volume as calculated from its fraction (12.5% of body weight), hematocrit (40%) and plasma density (1.025 g/mL) [23,24]. Water-soluble components were not analyzed in LLF, due to the high dilution factor of LLF (~10 mL) compared to the small amount of lung lining fluid. Abbreviations: ^†††^, *p* < 0.001 vs. liver; ^‡‡‡^, *p* < 0.001 vs. plasma; ^#^, *p* < 0.001 vs. PC precursors.

**Table 2:** Fractions of D9-Choline Metabolites of the Total Administered D9-Choline as Sum of All Analyzed Organs.

Time	Water-Soluble D9-Choline- Derived Metabolites	D9-Labeled Phospholipids	D3-Labeled Phospholipids
1.5 h	11.8 ± 1.5%	15.3 ± 1.4%	0.8 ± 0.1%
6 h	2.1 ± 0.3% ^†††^	10.6 ± 1.1% ^†††^	1.6 ± 0.1% ^†††^
24 h	1.0 ± 0.1% ^†††, ‡‡‡^	9.7 ± 1.1% ^†††^	2.5 ± 0.1% ^†††, ‡‡‡^

Tissues were extracted as described in Materials and Methods. Choline, its water-soluble metabolites, PC, lyso-PC and SPH were then analyzed with LC-MS/MS as described in Materials and Methods. Data are the sums of all water-soluble (A) or lipid (B) components derived from D9-choline metabolism and are indicated as the fractions of applied D9-choline (50 mg D9-choline chloride/kg body weight). Data are indicated as mean ± SE of 8–10 animals at the respective time points (1.5 h, 6 h, 24 h). Abbreviations: ^†††^, *p* < 0.001 vs. 1.5 h; ^‡‡‡^, *p* < 0.001 vs. 6 h.

## Data Availability

The data presented in this study are available on request from the corresponding author.

## Supplementary Materials

The following supporting information can be downloaded at: https://www.mdpi.com/article/10.3390/nu14030720/s1, Figure S1: Fractions of D3-labeled versus endogenous PC in the liver (A), plasma (B), lung (C), cerebrum (D) and cerebellum (E).; Figure S2: Composition of phosphatidylethanolamine (PE) subgroups in plasma and organs.
