# Supplementary material for: Choline Kinetics in Neonatal Liver, Brain and Lung—Lessons from a Rodent Model for Neonatal Care
Source: Nutrients. 2022 Feb 8;14(3):720. doi: 10.3390/nu14030720 (PMC8837973; doi:10.3390/nu14030720)
Supplement: Supplementary file 1 [file nutrients-14-00720-s001.zip › nutrients-1567091-supplementary.pdf]

Online supplement: Choline Kinetics in Neonatal Rat Liver, Plasma, Brain and Lung

Wolfgang Bernhard*, Marco Raith*, Anna Shunova*, Stephan Lorenz, Katrin Böckmann, Michaela Minarski, Christian F. Poets, Axel. R. Franz.

*: These authors equally contributed to the research

Affiliations: [1]Department of Neonatology, Children's Hospital, Faculty of Medicine, Eberhard-Karls-University, Tübingen, Germany.

Address of correspondence: Wolfgang Bernhard, MD, PhD, Department of Neonatology, Children's Hospital, Faculty of Medicine, Eberhard-Karls-University, Calwer Straße 7, D-72076 Tuebingen, Germany
Email: wolfgang.bernhard@med.uni-tuebingen.de
Phone: +49 7071 29 86377

Short title: Systemic neonatal choline kinetics

Conflict of interest statement: There are no conflicts of interest to declare

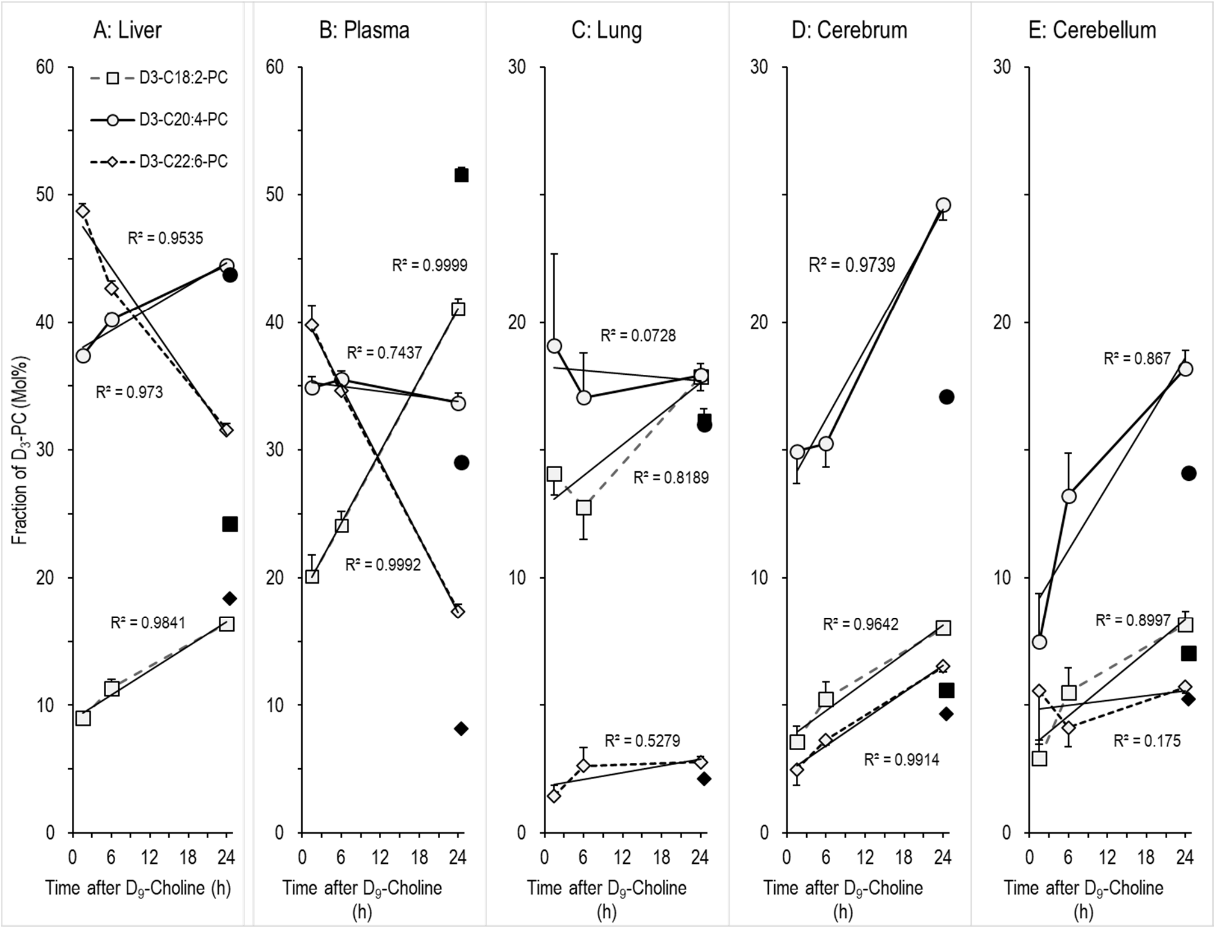

Figure S1: Fractions of D_3-labeled versus endogenous PC in liver (A), plasma (B), lung (C); cerebrum (D) and cerebellum (E). C18:2-PC, C20:4-PC and C22:6-PC represent the endogenous PC sub-groups containing a linoleic (C18:2n-6), arachidonic (C20:4n-6) or docosahexaenoic (C22:6n-3) acid residue, respectively, of all time points (N=27), whereas their D_8-choline-labeled analogues are means ± SE at the respective time points (N=8-10).

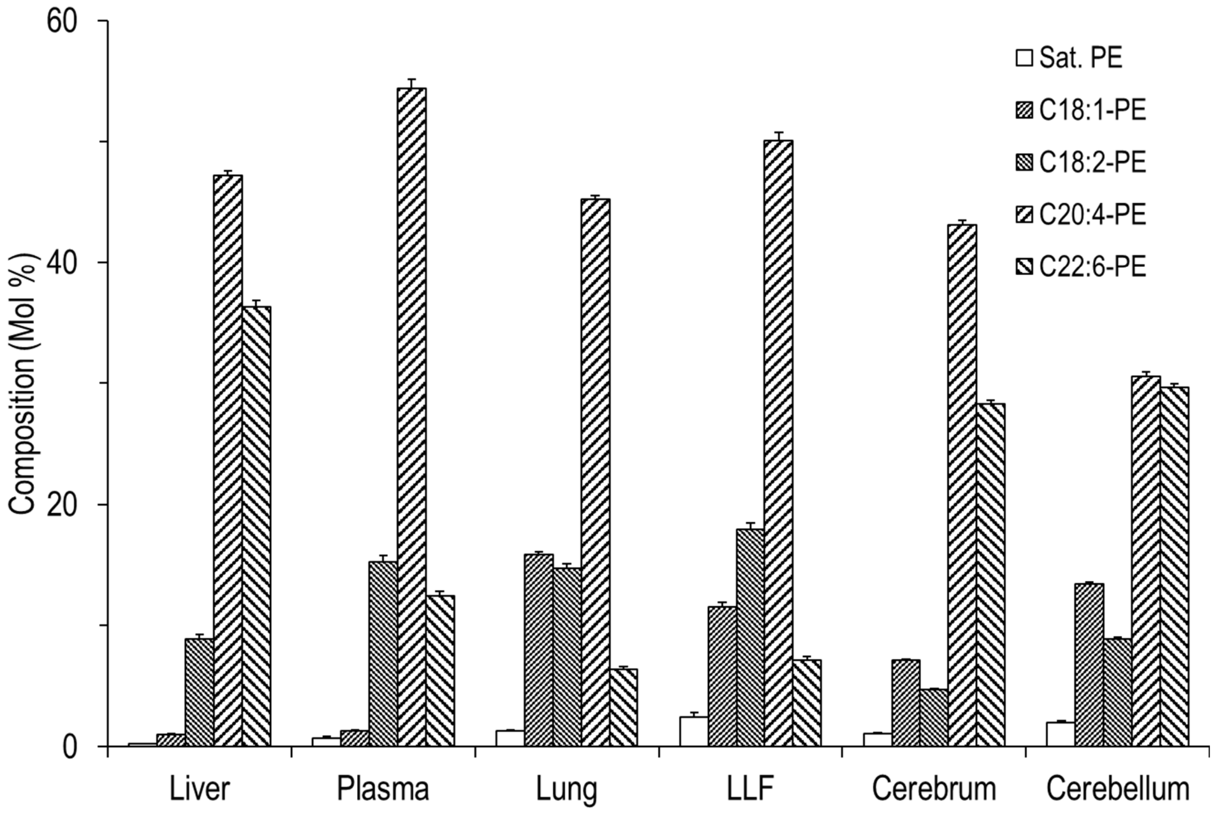

Figure S2: Composition of phosphatidylethanolamine (PE) subgroups in plasma and organs. PE molecular species were analyzed as indicated in Materials and Methods, and subgroup according to their content in 2 saturated fatty acid residues (Sat. PE) or a single oleic (C18:1-PE), linoleic (C18:2-PE), arachidonic (C20:4-PE) or docosahexaenoic (C22:6-PE) acid residue. Data are means±SE of N=27 experiments.
